# Analysis of transcriptome data reveals multifactor constraint on codon usage in *Taenia multiceps*

**DOI:** 10.1186/s12864-017-3704-8

**Published:** 2017-04-20

**Authors:** Xing Huang, Jing Xu, Lin Chen, Yu Wang, Xiaobin Gu, Xuerong Peng, Guangyou Yang

**Affiliations:** 1 Department of Parasitology, College of Veterinary Medicine, Sichuan Agricultural University, Chengdu, 611130 China; 2 Chengdu Agricultural College, Chengdu, 611130 China; 3 Meat-processing Application Key Laboratory of Sichuan Province, College of Pharmacy and Biological Engineering, Chengdu University, Chengdu, 610106 China; 4 College of Science, Sichuan Agricultural University, Ya’an, 625014 China

**Keywords:** *Taenia multiceps*, Codon usage pattern, Natural selection, Genome, Evolution

## Abstract

**Background:**

Codon usage bias (CUB) is an important evolutionary feature in genomes that has been widely observed in many organisms. However, the synonymous codon usage pattern in the genome of *T. multiceps* remains to be clarified. In this study, we analyzed the codon usage of *T. multiceps* based on the transcriptome data to reveal the constraint factors and to gain an improved understanding of the mechanisms that shape synonymous CUB.

**Results:**

Analysis of a total of 8,620 annotated mRNA sequences from *T. multiceps* indicated only a weak codon bias, with mean GC and GC3 content values of 49.29% and 51.43%, respectively. Our analysis indicated that nucleotide composition, mutational pressure, natural selection, gene expression level, amino acids with grand average of hydropathicity (GRAVY) and aromaticity (Aromo) and the effective selection of amino-acids all contributed to the codon usage in *T. multiceps*. Among these factors, natural selection was implicated as the major factor affecting the codon usage variation in *T. multiceps*. The codon usage of ribosome genes was affected mainly by mutations, while the essential genes were affected mainly by selection. In addition, 21codons were identified as “optimal codons”. Overall, the optimal codons were GC-rich (GC:AU, 41:22), and ended with G or C (except CGU). Furthermore, different degrees of variation in codon usage were found between *T. multiceps* and *Escherichia coli,* yeast, *Homo sapiens*. However, little difference was found between *T. multiceps* and *Taenia pisiformis*.

**Conclusions:**

In this study, the codon usage pattern of *T. multiceps* was analyzed systematically and factors affected CUB were also identified*.* This is the first study of codon biology in *T. multiceps*. Understanding the codon usage pattern in *T. multiceps* can be helpful for the discovery of new genes, molecular genetic engineering and evolutionary studies.

**Electronic supplementary material:**

The online version of this article (doi:10.1186/s12864-017-3704-8) contains supplementary material, which is available to authorized users.

## Background

The multiple codons that encode the same amino acid are defined as synonymous codons. The non-normal distribution of synonymous codon usage within and between genomes is termed codon usage bias (CUB) [1, 2]. Among the various factors that are known to dictate CUB in a variety of organisms, compositional constraints and translational selection are considered to be the main influences [3].

Studies of synonymous codon usage contribute to the understanding of the mechanisms of biased usage of synonymous codons [4], selecting suitable host expression systems [5], designing degenerate primers [6], predicting genes based on genomic sequences [7] and functional protein classification [8]. Synonymous CUB has been characterized in a number of organisms. However, the transcriptomes of only *Taenia pisiformis* [9] and *Taenia saginata* [10] have been reported from the *Taeniidae* family.

*Taenia multiceps* (*T. multiceps*) is a parasite found in nearly all regions of the world and causes coenurosis [11], which is not only associated with significant economic losses to the livestock industry, but also represents a threaten to human health [11–15].

In the present study, we analyzed the codon usage profile of *T. multiceps* from annotations of the transcriptome using the CodonW 1.4.2 program and multivariate statistical analysis. Knowledge of the codon usage pattern of *T. multiceps* is important in elucidating the mechanisms underlying synonymous CUB and also for improved *T. multiceps* genetic vaccine production through informed selection of the most suitable expression systems.

## Methods

### Sequence acquisition

A total of 20,896 annotated coding sequences (CDSs) were obtained from the adult *T. multiceps* transcriptome database (https://www.ncbi.nlm.nih.gov/bioproject/PRJNA80935/); all genes excluded gaps [16]. The codon usage of CDS from nuclear genome were analyzed, while all mitochondrial genes were excluded. Additionally, only genes greater than 450 base pairs (bp) were included to enhance the sequence quality in further analysis [9]. A total of 8,620 CDSs, including ribosomal genes (15 CDSs) and essential genes (15 CDSs), remained in the final analysis based on the CDS annotation information and the Database of Essential Genes (DEG) [17–21]. Some partial sequences are still referred to as “genes”.

### Indices of codon usage

The following codon indices were determined: relative synonymous codon usage (RSCU) [3], effective number of codons (ENc) [22, 23], codon adaptation index (CAI), and GC-content at the first, second and third codon positions (GC1, GC2 and GC3), frequency of either a G or C at the third codon position of synonymous codons (GC3s), and the average of GC1 and GC2 (GC12) [22].

**RSCU** is the ratio of the observed and expected codon frequencies under a uniform synonymous codon usage [3], with codon bias diminishing as this value approaches 1.0, while RSCU values exceeding 1.0 indicate higher than expected codon usage [3].

**ENc** indicates the magnitude of codon bias for individual genes. Over a range of values from 20 to 61[23], lower values indicate greater codon bias. Generally speaking, ENc values lower than 36 indicate strong codon bias [23, 24].

**CAI** values indicate the extent of bias toward codons in highly expressed genes. Over a range of values between 0 and 1.0, higher CAI values indicate higher expression and greater CUB [22, 25, 26]. The set of sequences used to calculate CAI values in this study were the genes coding for 15 ribosomal proteins in *T. multiceps* [23], so that it can provide an indication of gene expression level under the assumption that translational selection can optimize gene sequences according to their expression levels.

All the indices of the total number of genes analyzed are shown in Additional file 1.

### Principal Component Analysis (PCA)

Principal component analysis (PCA) have often been used to identify major trends of variation in synonymous codon usage among genes [27, 28]. In this paper, data were normalized in the manner developed by Sharp and Li [22] to define the relative adaptiveness of each codon. And then PCA based on the relative adaptiveness was applied to identify major trends of intragenomic variation in synonymous codon usage among genes [28]. In addition, we analyzed the distribution of PC scores for constitutively highly expressed genes (encoding ribosomal proteins) [28]. In each PC, the score for the *g*th gene (*y*_*g*_) was normalized by the mean (*m*) and the standard deviation (S.D.) of scores for all genes, expressed as *z*_*g*_ = (*y*_*g*_ - *m*)/S.D.. If the mean absolute *z* g score for the highly expressed genes was greater than 5.17 (an interval in which theoretically only 1.5% of all genes are included), then gene expression level (Expression) was identified as the main trend of variation in PC scores among genes.

### ENc-plot

The ENc-plot of ENc values plotted against GC3s values is used to analyze the influence of base composition on the codon usage in a genome [29]. A standard curve is generated to show the functional relationship between ENc and GC3 values under mutation pressure rather than selection pressure. In genes where codon choice is constrained only by a G + C mutation bias, predicted ENc values will lie on or around the GC3 curve. However, the presence of other factors, such as selection effects, causes the values to deviate considerably below the expected GC3 curve.

### PR2 bias plot

Parity rule 2 (PR2) plot analysis, which was also conducted to investigate CUB, is used to the impact of mutation and selection on codon usage [30]. This analysis is based on a plot of AT-bias [A3/(A3 + T3)] and GC-bias [G3/(G3 + C3)] at the third codon position of the four-codon amino acids in entire genes. The four-codon amino acids are alanine, arginine (CGA, CGT, CGG, CGC), glycine, leucine (CTA, CTT, CTG, CTC), proline, serine (TCA, TCT, TCG, TCC), threonine, and valine [31].

### Neutrality plots

Neutrality plots (GC12-GC3) [26] were used to evaluate the relationships among the three positions in *T. multiceps* codons. Following linear regression analysis, a slope of 0 indicates an absence of directional mutation pressure (complete selective constraints), while a slope of 1 indicates complete neutrality.

### Determination of optimal codons

Based on axis 1 ordination, the top and bottom 5% of genes were regarded as the high and low datasets, respectively. Codon usage in the two data sets was compared using chi square tests, with the sequential Bonferroni correction to assess significance [32]. Optimal codons were defined as those used at significantly higher frequencies (*P* < 0.01) in highly expressed genes compared with the frequencies in genes expressed at low levels [33].

### Software

The following programs were used in this study: Codon W (Ver.1.4.2) (http://sourceforge.net/projects/codonw/), CHIPS (http://emboss.sourceforge.net/apps/release/6.6/emboss/apps/chips.html), and CUSP (http://emboss.sourceforge.net/apps/release/6.6/emboss/apps/cusp.html). These programs were used to calculate CUB indices, such as GC, GC3s (G + C content at the third position of codons), and silent base compositions (A3s, T3s, C3s, and G3s, which indicate the frequency of codons with A, U, C, or G, respectively, at the synonymous third position). GRAVY, Aromo, RSCU and ENc values were also calculated and COA was performed.

## Results

### Codon composition analysis

**Fig. 1:**
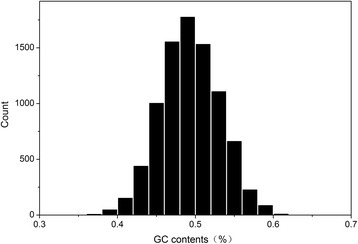
Distribution of GC contents in *T. multiceps* genes

As shown in Table 1 and Figure 1, the GC-content of the *T. multiceps* genes ranged from 36.5% to 62.1%. The GC-content of the total number of genes included in the analysis (8,620) were distributed mainly between 40% and 60%, with a mean value of 49.27%, indicating that a slight AT-rich bias in the genome. In addition, the average GC-content in the third codon position (GC3 = 51.43%, Table 1) was slightly higher than that among the total number (8,620) genes analyzed (49.27%).

**Table 1:** Mean values and standard deviation of GC, GC1, GC2, GC12, GC3, GC3s, GRVAY, Aromo, ENC and CAI values for reconstructed genes in *T.multiceps*

Class	Genes	Codons	GC (%)	GC1 (%)	GC2 (%)	GC12 (%)	GC3 (%)	GC3s (%)	Gravy	Aromo	ENC	CAI
Ribosome genes	15	5731	48.17 ± 3.38	54.62 ± 4.63	39.77 ± 4.11	47.19 ± 3.50	50.16 ± 5.10	48.55 ± 5.25	−0.54 ± 0.30	0.08 ± 0.03	57.39 ± 2.15	0.23 ± 0.03
Other genes	8605	2807063	49.27 ± 3.78	55.36 ± 4.79	41.01 ± 5.44	48.16 ± 3.98	51.43 ± 6.72	49.80 ± 6.83	−0.25 ± 0.39	0.08 ± 0.03	56.68 ± 3.41	0.23 ± 0.03
All genes	8620	2812794	49.27 ± 3.78	55.36 ± 4.79	41.01 ± 5.44	48.18 ± 3.98	51.43 ± 6.72	49.80 ± 6.83	−0.25 ± 0.39	0.08 ± 0.03	56.68 ± 3.41	0.23 ± 0.03

Note: G, guanine; C, cytosine; GC1, GC2, GC12 and GC3, GC content at the first, second, the average of GC1 and GC2 and third codon positions; GRAVY, grand average of hydropathicity; Aromo, aromaticity; ENc, effective number of codons; CAI, codon adaptation index

The ENc values among all 8,620 genes varied from 30.5 to 61, with an average of 56.68 (Table 1), and only two of these genes showed a high codon bias (ENc < 35). These results suggested that the existence of random codon usage in *T. multiceps*, with no strong CUB.

### Preferential codon usage

A shown in Table 2, frequent use of 32 of the 59 sense codons, including GCU, GCC and CGU, was observed. Furthermore, more than half (18/32) of the frequently used codons ended with G or C.

**Table 2:** Codon usage in *T. multiceps* genes

AA	Codon	N	RSCU	AA	Codon	N	RSCU
Ala	**GCU**	66571	1.26	Leu	**UUG**	54091	1.13
	**GCC**	60219	1.14		**CUU**	62280	1.30
	GCA	46898	0.89		**CUC**	66010	1.37
	GCG	37647	0.71		CUA	28125	0.59
Arg	**CGU**	41703	1.44		**CUG**	57257	1.19
	**CGC**	36670	1.27	Lys	AAA	66198	0.90
	**CGA**	34400	1.19		**AAG**	80146	1.10
	CGG	21406	0.74	Phe	UUU	56781	0.97
	AGA	20593	0.71		**UUC**	56843	1.03
	AGG	18942	0.65	Pro	**CCU**	38993	1.11
Asn	**AAU**	61374	1.07		**CCC**	38260	1.09
	AAC	53482	0.93		**CCA**	37916	1.08
Asp	**GAU**	83678	1.10		CCG	25730	0.73
	GAC	67932	0.90	Ser	**UCU**	41504	1.13
Cys	UGU	28391	0.99		**UCC**	43482	1.18
	**UGC**	29110	1.01		UCA	36776	1.00
Gln	CAA	51821	0.95		UCG	33005	0.89
	**CAG**	57603	1.05		AGU	35197	0.95
Glu	GAA	86515	0.92		AGC	31355	0.85
	**GAG**	101456	1.08	Thr	**ACU**	44571	1.17
Gly	**GGU**	56752	1.41		**ACC**	43688	1.14
	**GGC**	45893	1.14		ACA	36206	0.95
	GGA	37411	0.93		ACG	28553	0.75
	GGG	21005	0.52	Tyr	UAU	33694	0.82
His	CAU	31625	0.95		**UAC**	48161	1.18
	**CAC**	34908	1.05	Val	**GUU**	55062	1.19
Ile	**AUU**	65463	1.32		**GUC**	47059	1.02
	**AUC**	56412	1.14		GUA	23416	0.51
	AUA	26796	0.54		**GUG**	59224	1.28
Leu	UUA	20522	0.43				

Note: The preferentially used codons are displayed in bold

AA: amino acid; N: the number of codons

### Principal Component Analysis (PCA)

**Fig. 2:**
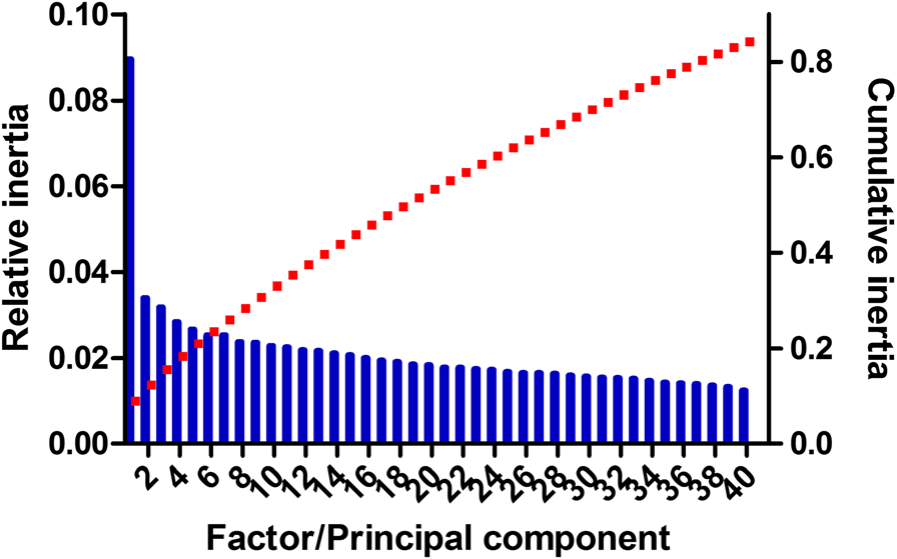
The relative (*blue bars*) and cumulative (*red squares*) inertia of the total of 40 factors from principal component analysis (PCA) using relative adaptiveness

Principal components (PCs) with variances greater than the maximal variance of the original variables were selected as the significant axes [27]. PCA based on the relative adaptiveness showed that the first principal component (PC1) explained 8.95% of the total variation, while the other three PCs accounted for 3.40%, 3.18% and 2.84% of the data (Fig. 2). Moreover, multivariable correlation analysis was performed to gain a better understanding of the relationship between relative codon bias and nucleotide composition (Table 3). As shown in table 3, there was a clear negative correlation between PC1 and GC, GC3 and GC3s (r = −0.693, −0.859and −0.865, respectively, *P* < 0.01), while PC1correlated positively with A3s or T3s (r = 0.643 and 0.304; *P* < 0.01). In addition, the ENc value correlated negatively with GC, GC1, GC2, GC3, G3s, C3s and GC3s (r = −0.217, −0.125, −0.43, −0.243, −0.063, −0.246 and −0.249, respectively; *P* < 0.01). These results suggested the ENc value decreased as the content of GC or GC3s increased, with a corresponding increase in the strength of codon bias. Furthermore, ENc values showed a significant positive correlation with the first and the second principal component (PC1 and PC2) (r = 0.230 and 0.204; *P* < 0.01). However, PC1explained a larger proportion of the variation at 8.95% (Fig. 2), indicating that the first axis is the major contributor to codon bias although other factors also have a strong influence on this parameter.

**Table 3:** Correlation coefficients between the positions of genes along the PC1, PC2 and the index of codon usage and synonymous codon usage bias among the total number of genes analyzed

	Length	GC	GC1	GC2	GC3	GC3s	A3s	T3s	G3s	C3s	Gravy	Aromo	CAI	FOP	ENc	PC1
Length																
GC	.064^**^															
GC1	.090^**^	.663^**^														
GC2	−0.007	.605^**^	.207^**^													
GC3	.050^**^	.724^**^	.239^**^	.063^**^												
GC3s	.055^**^	.738^**^	.264^**^	.076^**^	.995^**^											
A3s	-.063^**^	-.655^**^	-.280^**^	-.220^**^	-.728^**^	-.732^**^										
T3s	−0.016	-.613^**^	-.166^**^	-.136^**^	-.805^**^	-.808^**^	.264^**^									
G3s	.059^**^	.303^**^	.207^**^	-.249^**^	.564^**^	.570^**^	-.312^**^	-.409^**^								
C3s	.026^*^	.535^**^	.072^**^	.061^**^	.802^**^	.801^**^	-.592^**^	-.655^**^	.033^**^							
Gravy	-.047^**^	−0.009	-.209^**^	−0.007	.140^**^	.121^**^	-.319^**^	-.110^**^	-.278^**^	.242^**^						
Aromo	-.099^**^	-.265^**^	-.486^**^	-.225^**^	.080^**^	.041^**^	-.076^**^	0.015	-.185^**^	.265^**^	.440^**^					
CAI	.074^**^	.210^**^	.124^**^	-.045^**^	.302^**^	.306^**^	-.377^**^	0.002	.070^**^	.415^**^	-.075^**^	.055^**^				
FOP	.056^**^	.397^**^	.141^**^	.177^**^	.426^**^	.431^**^	-.445^**^	-.227^**^	-.034^**^	.564^**^	.007	.035^**^	-.769^**^			
ENc	0.017	-.217^**^	-.125^**^	-.043^**^	-.243^**^	-.249^**^	.337^**^	.057^**^	-.063^**^	-.246^**^	-.039^**^	0.021	-.310^**^	-.254^**^		
PC1	-.073^**^	-.693^**^	-.273^**^	-.142^**^	-.859^**^	-.865^**^	.643^**^	.704^**^	-.413^**^	-.749^**^	-.083^**^	-.026^*^	-.321^**^	-.433^**^	.230^**^	
PC2	-.092^**^	-.062^**^	-.127^**^	-.078^**^	.048^**^	.040^**^	.444^**^	-.438^**^	.249^**^	-.108^**^	-.070^**^	0.008	-.358^**^	-.352^**^	.204^**^	0

Note: ** *P* < 0.01. * *P* < 0.05

**Fig. 3:**
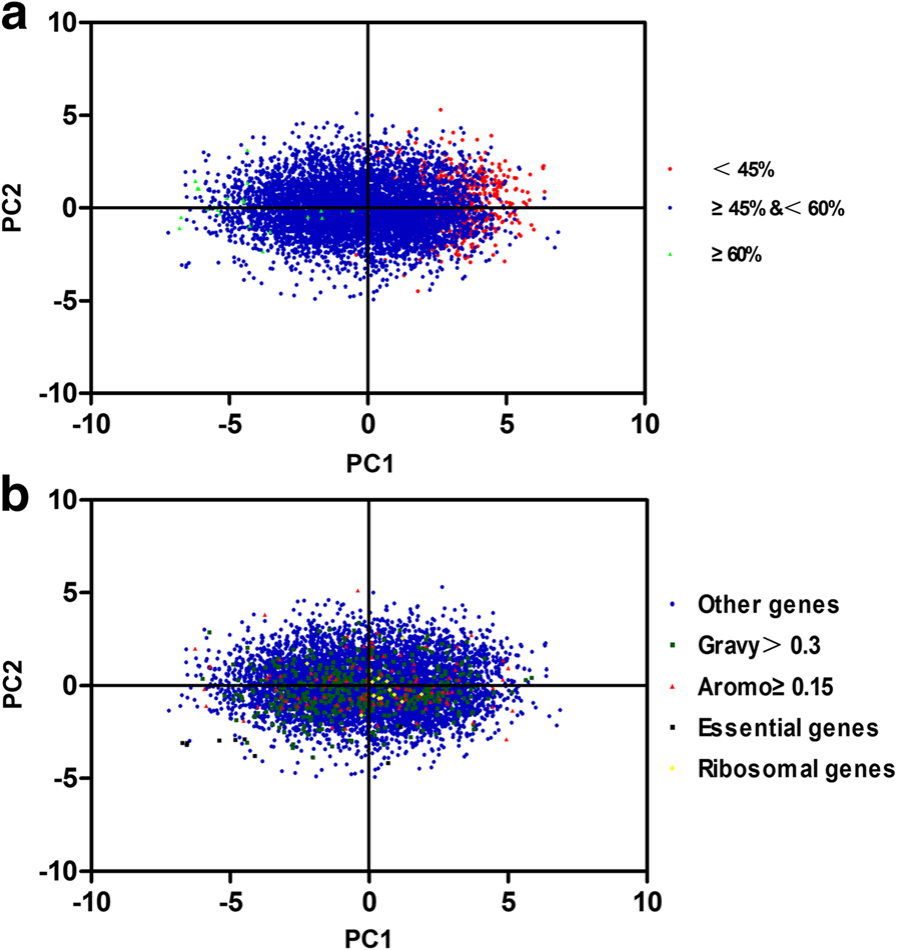
Principal component analysis of relative synonymous codon usage in *T. multiceps* genes. The distribution of genes is shown along PC1 and PC2. A. *Red dots*, *blue squares* and *green triangles* indicate genes with G + C contents of less than 45%, more than or equal to 45%, but less than 60% and more than or equal to 60%, respectively. B. *Blue dots*, *light green squares*, *red triangles*,* black squares* and *yellow squares* indicate other genes, genes with a GRAVY value higher than 0.3, genes with an Aromo value greater than or equal to 0.15, essential genes and ribosomal genes

The GC-content of each gene was then investigated in terms of the codon usage preference. Following classification according to GC-content (GC < 45%, 45% ≤ GC < 60%, and GC ≥ 60%), all the genes were then marked along the first two PCs (Fig. 3A). The genes with GC <45% and GC ≥ 60% were distributed mainly to the right and left of PC1, respectively, while the genes with GC contents ranging from 45% to 60% were clustered in the center of the plot.

To characterize the codon usage patterns of the different kinds of gene, hydrophobic genes with scores >0.3, aromatic genes with scores ≥0.15, essential genes and ribosomal genes were selected from the 8,620 genes included in this study. The distribution of these genes was marked along PC1 and PC2 based on the principal component analysis (Fig. 3B). A majority of the ribosomal genes were clustered to the right of PC1, while essential genes to the left of PC1. Hydrophobic genes with scores >0.3, aromatic genes with scores ≥0.15 and other genes were located mainly in the central region of PC1.

These results suggested that compositional constraints are the main factor accounting for the CUB, although other factors are also strong influences.

### Relationship between ENc and GC3s

**Fig. 4:**
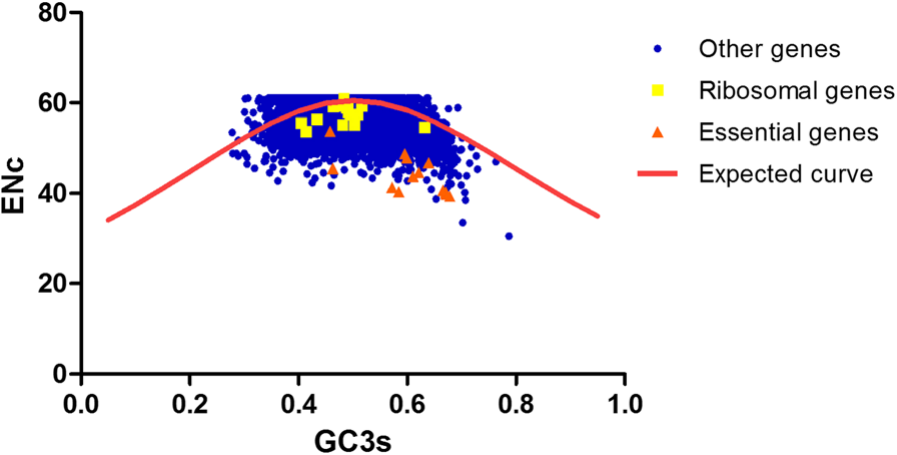
Distribution of effective number of codons (ENc) and GC3s of *T. multiceps* genes. The *solid line* (*red*) indicates the expected ENc value if the codon bias is only due to GC3s. The *yellow squares*, *orange triangles* and *blue dots* indicate ribosomal genes, essential genes and other genes, respectively

The features of codon usage among genes can be visualized by plotting ENc against GC3s [9]. As shown in Figure 4, a majority of *T. multiceps* genes were located under the curve of expected ENc values, while only a small number were distributed along or above. This implied that conditional mutations exert only weak influences on CUB of *T. multiceps*, although a major role may be played by other factors, such as natural selection. Most ribosomal genes were scattered along the expected ENc curve, while all essential genes were located at a marked distance below. These results implied that the CUB of ribosomal genes was affected mainly by mutations, while that of essential genes was influenced mainly by selection.

**Fig. 5:**
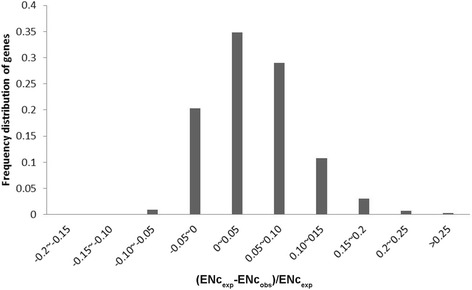
Frequency distribution of (ENc_exp_-ENc_obs_)/ENc_exp_, where ENc is the effective number of codons and “exp” and “obs” indicate the expected and observed values, respectively

To a gain a more intuitive understanding of the difference between the observed and expected ENc values, the frequency distribution of (ENc_exp_-ENc_obs_)/ENc_exp_, (ENc_exp_-ENc_obs_)/ENc_exp_ was plotted (Fig. 5). Most genes had (ENc_exp_-ENc_obs_)/ENc_exp_ values ranging from −0.05 to 0.2, with a peak in the distribution of values between 0–0.05. The significant differences observed between the observed and expected ENc values indicated that mutation exerts only a weak effect in shaping CUB.

### PR2 bias plot analyses

**Fig. 6:**
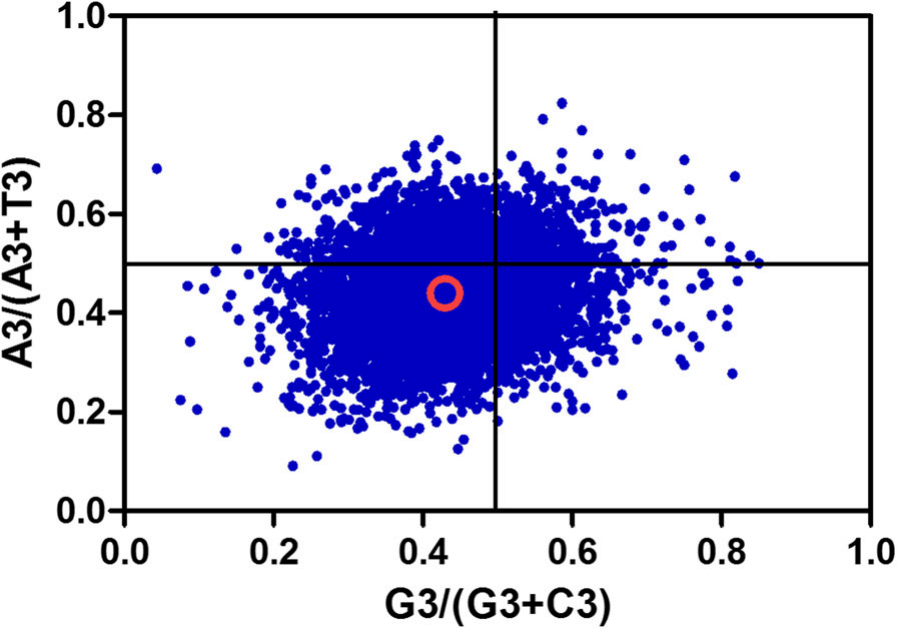
PR2-bias plot [A3/(A3 + T3) against G3/(G3 + C3)]. *Red* open circle indicates the average position for each plot. Average position coordinates are x = 0.4296 ± 0.0846, y = 0.4340 ± 0.0860

PR2 plot analysis was conducted assess to the impact of mutation and selection on CUB (Fig. 6). In this analysis, most genes were distributed in the lower left quadrant of the PR2-plot (Fig. 6), implying that C and T (pyrimidines) were used more frequently than G and A (purines) in *T. multiceps* codons. These data provide further evidence that factors other than mutational pressure, such as natural selection, also contribute to CUB.

### Neutrality plot analysis

**Fig. 7:**
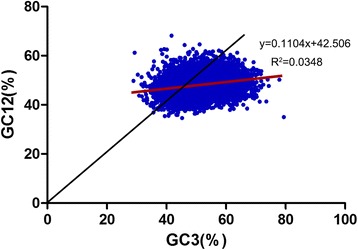
Neutrality plot analysis of the GC12 and GC3 values of the *T. multiceps* transcriptome*.* GC12 represents for the average value of GC-content in the first and second position of the codons (GC1 and GC2), while GC3 represents the GC-content in the third position. The red line shows the linear regression of GC12 against GC3, *R* ^2^ = 0.0348, *P* < 0.01. OP = 47.78, where OP is the intersection of the regression curve and the diagonal, and represents the point at which GC12 equals GC3

In the neutrality plot of all the genes generated to evaluate the relationships among the three positions in *T. multiceps* codons (Fig. 7), most did not lie on or along the diagonal line. In addition, the ranges of GC12 and GC3 were narrow (0.3464–0.6818 and 0.2893–0.7944, respectively). These data suggested that *T. multiceps* codon usage is affected by natural selection. Moreover, linear regression of the entire coding sequence data yielded a slope of 0.1104, revealing that directional mutation pressure accounts for only 11.04% of the effect, while other factors (e.g. natural selection) account for 88.96% of the influence [34, 35]. Additionally, a significant positive correlation was identified between GC12 and GC3 (r = 0.187, *P* < 0.01), suggesting the influence of directional mutation pressure at all codon positions and that codon usage was affected by mutation.

### Gene expression level and synonymous CUB

To explore the relationship between gene expression level and codon preference, we calculated the coefficients of the correlations between the codon adaptation index (CAI) and other gene features, including nucleotide composition and ENc values (Table 3). As shown in Table 3, CAI showed significant negative correlations with ENc value, PC1, PC2, GC2, A3s, and GRAVY value (r =−0.321,−0.358,−0.333,−0.045,−0.377, and−0.075, respectively; *P* < 0.01). However, CAI showed significant positive correlations with gene length and the other nucleotide composition indices (GC, GC1, GC3, GC3s, G3s, C3s and Aromo) (r = 0.074, 0.210, 0.124, 0.302, 0.306, 0.070 and 0.415, respectively; *P* < 0.01). These results indicated that the codon usage in *T. multiceps* was affected by gene expression levels. To be more specific, genes with higher expression levels had a greater degree of CUB and GC-rich content. Furthermore, these genes exhibited preference for codons with C or G at the synonymous position. Based on these results, we deduced that both nucleotide composition and gene expression levels play important roles in *T. multiceps* codon usage.

The relationship between amino-acid composition index and CUB in *T. multiceps* was investigated by Spearman’s rank correlation analysis to determine the correlation coefficients of the positions of the genes along the first two PCs with the corresponding amino-acid usage indices (Table 4).

**Table 4:** Correlation coefficients between the positions of genes along the PC1, PC2 and index of amino acid usage among the total number of genes analyzed

	PC1	PC2	Gravy	Aromo
PC2	.000			
Gravy	-.032^**^	.509^**^		^*^
Aromo	-.051^**^	.637^**^	.440^**^	
CAI	.081^**^	.001	-.075^**^	.055^**^

Note: ** *P* < 0.01

**Fig. 8:**
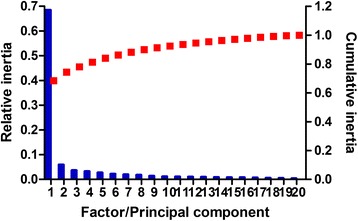
The relative (*blue bars*) and cumulative (*red squares*) inertia of the total 20 factors from principal component analysis (PCA) of the amino-acid usage frequencies

In the principal component analysis, the first two PCs generated accounted for 74.35% of the variation in amino-acid usage. PC1 explained 68.43% of the variation in amino-acid usage (Fig. 8), with the genes showing significant negative correlations with GRAVY score and Aromo value score (r =−0.688 and−0.454, respectively; *P* < 0.01), while a significant positive correlation was identified with CAI (r = 0.081, *P* < 0.01). PC2 explained 5.92% of the variation, and showed significant positive correlations with GRAVY score and Aromo value (r = 0.509 and 0.637, respectively; *P* < 0.01). In contrast to the results for *E. coli* [36] and *B. mori* [35], CAI was found to be the most important factor influencing the amino-acid usage of *T. multiceps*, with aromaticity having the second most important influence, followed by hydrophobicity.

### Effect of the hydrophobicity and aromaticity of encoded protein on codon bias

We also investigated the influence of other factors on codon usage in *T. multiceps* genes. A correlation analysis was performed to evaluate whether GRAVY and Aromo values were related to nucleotide composition and ENc values. As shown in Table 3, GRAVY values of the encoded proteins showed significant negative correlations with GC1, A3s, T3s, G3s and ENc values (r =−0.209,−0.319,−0.110,−0.278, and−0.039, respectively; *P* < 0.01), while this value showed significant positive correlations with GC3, GC3s, and C3s (r = 0.140, 0.121, 0.242, respectively; *P* < 0.01). In addition, Aromo values of the encoded proteins showed significant negative correlations with GC, GC1, GC2, A3s and G3s (r =−0.265,−0. 486,−0. 225,−0. 076, and−0. 185, respectively; *P* < 0.01). This indicated that codon usage variations were associated with both the degree of hydrophobicity and aromatic among the amino acids.

### Gene length and synonymous CUB

According to Table 3, gene length showed no significant correlation with ENc values (r = 0.017, *P* > 0.05), but showed significant positive correlations with GC1, GC3, GC3s, C3s, G3s, and CAI (r = 0.090, 0.050, 0.055, 0.026, 0.059 and 0.074, respectively; *P* < 0.01). These results suggested an absence of any significant correlation between gene length and the CUB, although relatively higher expression of the longer genes was observed.

### Optimal translational codons

Twenty-one codons, including UUC, CUC, CUG and AUC, were identified as optimal translational codons based on the average RSCU values of the high and low datasets. Of these, the AU/GC ratio was 41:22, and the optimal codons (except CGU) all ended with G or C (Table 5).

**Table 5:** Translational optimal codons of *T. multiceps*

AA	Codon	High RSCU(N)	Low RSCU(N)	AA	Codon	High RSCU(N)	Low RSCU(N)
Phe	UUU	0.78(2687)	1.13(1612)	Ser	UCU	1.01(1455)	1.14(1901)
	UUC*	1.22(4172)	0.87(1229)		UCC*	1.54(2224)	1.01(1678)
Leu	UUA	0.25 (555)	0.63(1404)		UCA	0.75(1079)	1.16(1937)
	UUG	0.79(1723)	1.41(3125)		UCG	0.90(1294)	0.83(1389)
	CUU	1.10(2413)	1.32(2910)		AGU	0.85(1231)	1.07(1785)
	CUC*	2.03(4437)	0.84(1864)		AGC	0.95(1372)	0.78(1295)
	CUA	0.50(1096)	0.62(1375)	Pro	CCU	1.05(1557)	1.15 (985)
	CUG*	1.33(2917)	1.17(2592)		CCC*	1.37(2037)	0.82 (703)
Ile	AUU	1.06(2541)	1.42(2609)		CCA	0.90(1336)	1.36(1163)
	AUC*	1.52(3644)	0.87(1605)		CCG	0.69(1026)	0.67 (575)
	AUA	0.42(1019)	0.70(1292)	Thr	ACU	1.02(1663)	1.16(1879)
Met	AUG	1.00(2622)	1.00(2746)		ACC*	1.50(2449)	0.92(1492)
Val	GUU	0.90(2027)	1.37(2157)		ACA	0.77(1262)	1.08(1756)
	GUC*	1.26(2837)	0.79(1249)		ACG	0.71(1153)	0.85(1380)
	GUA	0.42 (948)	0.63 (989)	Ala	GCU	1.09(2436)	1.27(3404)
	GUG*	1.42(3213)	1.20(1890)		GCC*	1.40(3133)	0.96(2575)
Tyr	UAU	0.59(1234)	1.05(1239)		GCA	0.75(1686)	1.04(2780)
	UAC*	1.41(2920)	0.95(1124)		GCG	0.76(1691)	0.73(1955)
His	CAU	0.80(1079)	1.10(1275)	Cys	UGU	0.87(1565)	1.11 (806)
	CAC*	1.20(1630)	0.90(1052)		UGC*	1.13(2036)	0.89 (649)
Gln	CAA	0.86(1319)	1.01(3748)	Trp	UGG	1.00(1904)	1.00 (714)
	CAG*	1.14(1755)	0.99(3698)	Arg	CGU*	1.52(1377)	1.28(2285)
Asn	AAU	0.87(1721)	1.21(3282)		CGC*	1.61(1460)	1.03(1841)
	AAC*	1.13(2251)	0.79(2132)		CGA	1.15(1040)	1.10(1972)
Lys	AAA	0.79(1230)	0.95(5661)		CGG	0.78 (707)	0.72(1286)
	AAG*	1.21(1865)	1.05(6277)		AGA	0.46 (421)	1.01(1808)
Asp	GAU	0.91(1832)	1.20(4650)		AGG	0.47 (429)	0.85(1523)
	GAC*	1.09(2215)	0.80(3082)	Gly	GGU	1.34(2761)	1.37(1483)
Glu	GAA	0.70(1403)	1.01(8782)		GGC*	1.41(2917)	0.92 (997)
	GAG*	1.30(2606)	0.99(8660)		GGA	0.81(1665)	1.03(1110)
					GGG	0.44 (919)	0.68 (739)

Note: AA: amino acid; N: number of codons; RSCU: Relative synonymous codon usage. The codon usage of 431 genes (5% of the total number of genes) from the extremes of the principal were pooled. The codon usage of both pools was compared using Chi squared test, to identify optimal codons. Asterisks denote codons that occurred significantly more often (*P* < 0.01)

**Fig. 9:**
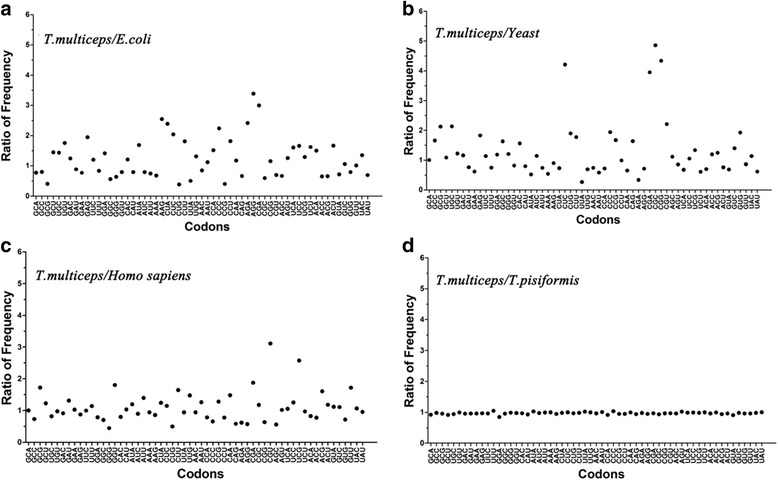
**A**-**D** Comparisons in the ratio of codon usage frequency (1/1000) of *T. multiceps* relative to *E. coli*, yeast, *H. sapiens* and *T. pisiformis*, respectively. Ratios higher than 2 or lower than 0.5 indicate differences in the codon usage preference between the two species

Closely-related species always have similar patterns of codon usage, while distantly related organisms, such as *Escherichia coli, Saccharomyces cerevisiae* and *Drosophila melanogaster* possess quite different codon usage patterns [37]. It is generally acknowledged that a ratio of codon usage frequency between two species that is greater than 2, or less than 0.5 indicates distinct CUB [38], while a ratio between these two values indicates a close codon usage preference. The ratios of codon usage frequency of *T. multiceps* compared with the four model organisms *E. coli*, *S. cerevisiae*, *Homo sapiens* and *T. pisiformis*, showed that number of codons with ratios greater than 2 or less than 0.5 was 10, 9, 4 and 0, respectively. This suggested that a relatively greater variation in codon preferences between *T. multiceps* and *E. coli*, *S. cerevisiae*, or *Homo sapiens* than that between *T. multiceps* and *T. pisiformis*, indicating that closer relationships between species are associated with less variation in codon usage (Fig. 9).

## Discussion

Nucleotide composition is considered to be one of the most important factors that shapes codon usage among genes and genomes, with GC-content reflecting the overall trend of codon mutation [31]. The average GC-content of the total of 8,620 *T. multiceps* genes investigated was 49.27% (slightly below the average AT content), while the average GC3 content was slightly higher at 51.43%. These results are consistent with the GC and AT contents of *Giardia lamblia* [39] and *T. saginata* [10].

The average effective number of codons (ENc) among the *T. multiceps* genes was 56.68, with only two genes showing a strong CUB (ENc < 36). This indicates random codon usage in *T. multiceps*, with no strong codon bias, which is in accordance with the pattern in *B. mori* [35]*.* Furthermore, more than half of the high frequency codons ended with G/C (18/32); this phenomenon has been found in many other GC-rich organisms, including bacteria, archaea, fungi, wheat and rice [40–43].

CUB is a complex evolutionary phenomenon known to exist in a wide variety of organisms, including prokaryotes, as well as unicellular and multicellular eukaryotes [10]. Numerous hypotheses have been proposed to explain this phenomenon including the neutral theory [44] and the selection-mutation-drift balance model [3]. The number of factors reported to affect CUB is increasing, with gene length [45], GC-content [46, 47], recombination rate [46, 48–50], and gene expression level [45, 48, 51] shown to exert influences. Other studies have shown that RNA and protein structure [29, 52–54], intron length [55], population size [56], evolutionary age of the genes [57], and environmental stress [58], in addition to the hydrophobicity and the aromaticity of the encoded proteins [59, 60] are influencing factors. In this study, various factors such as gene compositional constraints, mutation pressure, gene expression level and, in particular natural selection, were all found to contribute to shaping the codon usage of *T. multiceps.* Other factors, such as hydrophobicity and aromaticity of the encoded proteins were implicated in generating the CUB of *T. multiceps*, while our analysis indicated that amino-acid selection also affects translational efficiency of *T. multiceps*.

Base changes in first and second positions of the codon lead to changes in the encoded amino-acid sequence, while the third codon position rarely induces such sequence variation. It is generally acknowledged that the third codon position is subject to lower selection pressure compared with that of the first and second codon positions. Thus, ENc-GC3s correlation analysis, PR2 bias plot analyses and neutrality plot analysis based on GC3 or GC3s are vitally important for elucidation of the CUB patterns in many organisms.

ENc-GC3s correlation analysis showed that mutation plays a minor role in shaping CUB in *T. multiceps,* while other factors, such as natural selection, exert significant effects on CUB in this species. Additionally, correlation analysis indicated that the CUB of ribosomal genes was shaped mainly by mutations, while essential genes were affected mainly by natural selection. Further evidence in support of this conclusion was provides by the PR2 bias plot analyses, which also indicated that selection is the major factor that shapes CUB in *T. multiceps*. ENc plots provide a method of quantifying the CUB of synonymous codons; however, this analysis alone is insufficient for determining the exact contributions of natural selection and mutational pressure to CUB within a species [35, 61]. In this study, we generated a neutrality plot to provide more precise information on this issue. According to the neutrality plot, directional mutation pressure accounts for only 11.04% of the effect, while other factors, such as natural selection, account for 88.96% [34, 35]. Therefore, natural selection was thought to be the major factor affecting the codon usage variation in *T. multiceps*. These results are similar to those obtained in investigations of *B. mori* [35].

Natural selection can enhance efficiency of transcription/translation by preferential usage of alternative synonymous codons. The study of Drosophila and Caenorhabditis revealed that significant codon usage bias was existed in highly expressed genes, and this is due to the increased effectiveness and accuracy during translation by preferential usage of optimal synonymous codons [45, 62]. Since synonymous mutations do not change the final protein product, selection for optimal codons is thought to be fairly weak [63]. This explains the possible relation between natural selection and the overall low levels of CUB in *T. multiceps*.

Previous studies have revealed that CUB in mammals is not correlated with the gene expression levels. However, in *Arabidopsis thaliana* [64], *Oryza sativa* [65], *C. elegans* [45], *B. mori* [35] and *T. saginata* [10], genes expressed at relatively high levels exhibited a greater degree of CUB.Various analyses can be used to assess gene expression levels, including EST (expressed sequence tag) counting [66], CAI values [10, 45] and ENc values [67]. In this study, calculation of CAI values was adopted to evaluate the levels of expression of *T. multiceps* genes. CAI and ENC values showed a significant negative correlation with PC1, suggesting gene expression levels influence CUB in *T. multiceps*, with stronger CUB in highly expressed genes.

For various organisms, such as *Populus tremula* [68], *Caenorhabditis elegans* [45], *Drosophila melanogaster* [47], *Arabidopsis thaliana* [45] *Silene latifolia* [69] and *T. saginata* [10], significant negative correlations were found between gene length and CUB. To account for this phenomenon, Moriyama and Powell proposed that selection constraints tend to reduce the length of highly expressed genes to generate shorter proteins that perform functions similar to those of longer proteins; thus reducing the energy expenditure required to generate a protein with a specific function [70]. In *T. multiceps,* however, gene length was found to be irrelevant in shaping CUB, although it was positively correlated with the gene expression level. This finding is inconsistent with that obtained in studies of *T. saginata* [10] and further investigations are required to explore the mechanisms of this phenomenon.

Identification of optimal codons could provide valuable information for use in molecular genetics studies of evolutionary and rational rearrangement (transformation) of codon usage [71–73]. Under normal circumstances, the optimal codons tend to reflect the GC and AT content of the genomes [43, 74], such as those of bacteria, archaebacteria and fungi. In the present study, the GC-content of codons in the *T. multiceps* transcriptome was lower than the AT content (GC:AT, 0.97:1), although 21 optimal codons found to be GC-rich (AU:GC, 41:22), with most ending in G/C. The same phenomenon has been reported in other organisms, such as *Populus tremula* (average GC-content, 45%) [68], *Drosophila* (average GC-content, 35%) [75], *T. pisiformis* (average GC-content, 49.48%), and *T. saginata* (average GC-content, 43.61%), with most favored codons being GC-rich or ending with G and/or C. In *Triticum aestivum* [76], *Hordeumvulgare* [61], *Oryza sativa* [65] and *Zea mays* [77], the average GC-content is 55.6%, 59.3%, 56.8% and 60% respectively, with optimal codons being AT-rich or ending in G or C.

Correspondence analysis (COA) is widely used to elucidate the variation in synonymous codon usage among genes. However, COA based on RSCU can be affected by biases such as amino acid biases [78]. Principal Component Analysis (PCA) using relative adaptiveness [28] or within-block correspondence analysis [79] can avoid the biases. Thus in this paper, PCA using relative adaptiveness was adopted to perform multivariate analysis other than correspondence analysis.

## Conclusions

Our analysis of the codon usage pattern of *T. multiceps* indicates that natural selection is the major factor influencing the codon usage variation in this species, while other factors such as nucleotide composition, mutational pressure, and gene expression level, also contribute to shaping the CUB. Furthermore, we identified 21 optimal codons, all of which ended in G/C.

In summary, our analysis further elucidates the codon usage pattern in *T. multiceps*, and provides the basis of further investigations for the identification of novel genes, as well as molecular genetic engineering and evolutionary studies in this species.

## Additional files

Additional file 1: All the indices of the total number of genes analyzed. (XLS 3493 kb)

## Abbreviations

Aromo: aromaticity
CAI: the codon adaptation index
CDSs: coding sequences
CUB: codon usage bias
DEG: essential genes
ENc: the effective number of codons
GC1: the GC-content at the first codon positions
GC12: the average of GC1 and GC2
GC2: the GC-content at the second codon positions
GC3: the GC-content at the third codon positions
GC3s: the frequency of either a guanine or cytosine at the third codon position of synonymous codons
GRAVY: grand average of hydropathicity
PC1: the first principal component
PC2: the second principal component
PCA: principal component analysis
PCs: Principal Components.
PR2: parity rule 2
RSCU: the relative synonymous codon usage
